# Transdermal Delivery of α-Aminophosphonates as Semisolid Formulations—An In Vitro-Ex Vivo Study

**DOI:** 10.3390/pharmaceutics15051464

**Published:** 2023-05-11

**Authors:** Dorottya Kocsis, Petra Regina Varga, Rusul Keshwan, Mina Nader, Miléna Lengyel, Pál Szabó, István Antal, Károly Kánai, György Keglevich, Franciska Erdő

**Affiliations:** 1 Faculty of Information Technology and Bionics, Pázmány Péter Catholic University, Práter u. 50a, H-1083 Budapest, Hungary; 2 Department of Organic Chemistry and Technology, Budapest University of Technology and Economics, H-1521 Budapest, Hungary; 3 Department of Pharmaceutics, Semmelweis University, H-1092 Budapest, Hungary; 4 Centre for Structural Study, Research Centre for Natural Sciences, H-1117 Budapest, Hungary

**Keywords:** α-aminophosphonates, transdermal penetration, topical formulation, skin-on-a-chip, rheology, droplet size distribution

## Abstract

α-Aminophosphonates are organophosphorus compounds with an obvious similarity with α-amino acids. Owing to their biological and pharmacological characteristics, they have attracted the attention of many medicinal chemists. α-Aminophosphonates are known to exhibit antiviral, antitumor, antimicrobial, antioxidant and antibacterial activities, which can all be important in pathological dermatological conditions. However, their ADMET properties are not well studied. The aim of the current study was to provide preliminary information about the skin penetration of three preselected α-aminophosphonates when applying them as topical cream formulations in static and dynamic diffusion chambers. The results indicate that aminophosphonate **1a**, without any substituent in the para position, shows the best release from the formulation and the highest absorption through the excised skin. However, based on our previous study, the in vitro pharmacological potency was higher in the case of para-substituted molecules **1b** and **1c**. The particle size and rheological studies revealed that the 2% cream of aminophosphonate **1a** was the most homogenous formulation. In conclusion, the most promising molecule was **1a**, but further experiments are proposed to uncover the possible transporter interactions in the skin, optimize the topical formulations and improve PK/PD profiles in case of transdermal delivery.

## 1. Introduction

**Scheme 1:**
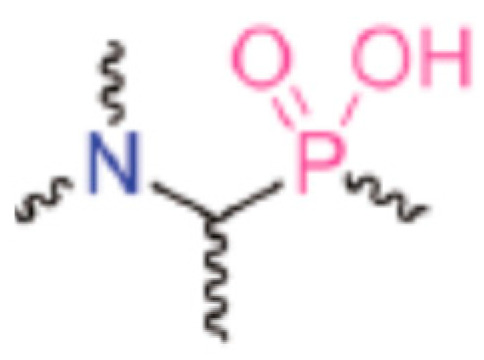
α-Aminophosphonic acid basic structure.

α-Aminophosphonic acids can be considered analogues of amino acids, in which the carboxylic group is replaced by a phosphonic acid (or a phosphinic acid) function [1]. They all include the characteristic N-C-P scaffold (Scheme 1).

The natural α-aminophosphonates and their biological activity were discovered more than 60 years ago [2,3]. The chemistry and the research on the biological activity of these compounds are continuously developing and makes up a distinct branch of phosphorus chemistry [4]. α-Aminophosphonates (AAPs) are able to influence several physiologic and pathologic processes, and they have a broad spectrum of applications from agrochemistry to medicine. A few of these compounds have already been marketed. Various aspects of their biological activities in natural systems are reviewed [5,6,7,8,9,10,11,12,13]. The biological effects of α-aminophosphonates are usually realized through enzyme inhibition. The enzyme inhibitory properties of these molecules are still being extensively researched and are a focus in medicinal chemistry. The N-C-P molecular fragment offers a broad spectrum of possibilities for structural modifications, which may lead to good biological relevance. Although carboxylic and phosphonic acid groups differ in shape (tetrahedral at phosphorus versus planar at carbon), acidity (phosphonic acid is more acidic) and steric bulk (the phosphorus atom has a much larger atomic radius than carbon), α-aminophosphonic and phosphinic acids are often considered simple analogues of their natural counterparts, carboxylic acids.

The facilitated membrane transport of the zwitterionic aromatic α-amino acids [14] and alanine [15] by α-aminophosphonates has been described earlier. APPs serve as carriers in the transport of amino-acids and the hydrophilic model drug alanine through the lipid membrane barrier. The parameters such as Caco-2 (colon adenocarcinoma) cell permeability, MDCK (MadinDarby canine kidney) cell permeability, BBB (blood-brain barrier) penetration, HIA (human intestinal absorption), skin permeability and plasma protein binding ability of amino phosphonate derivatives were predicted in silico by PreADMET online software [16,17,18]. The skin permeability studies have two purposes: this is a crucial parameter that can define the transdermal delivery of the compound as the risk assessment during accidental contact with the skin, and on the other hand, it can be important as a drug delivery route for treatment of dermatological diseases, where the target site is the skin tissue itself (e.g., melanoma, dermal infections, wound, sunburn, different types of dermatitis, psoriasis, etc.). Based on the anticancer [19,20,21], antioxidant, free radical scavenger, antifungal [22] and antibacterial [23] effects of APPs, which have been reported by different authors, it can be assumed that these compounds may have a beneficial therapeutic effect in the above-mentioned skin disorders.

APPs can be recognized by various enzymes or receptors as false substrates or inhibitors. The target enzymes of the topically applied APPs can be the neutral endopeptidase (NEP) expressed in different cancer cells. APPs act as inhibitors of the proteases implicated in cancerogenesis [19], including skin malignancies. Thus, the target site for APPs’ delivery can be the dermal barrier itself. It has also been reported that APPs are human glucokinase activators, which suggests their anti-diabetic potential [18]. Impaired wound healing and other skin abnormalities might appear and should be treated in instances of diabetic complications.

As it is shown above, the biological activity of this versatile group of P-containing organic compounds has been extensively studied, and the new results and observations influence their further exploration in medicinal chemistry. However, less is known about the capability of this class of molecules to cross biological barriers, such as the skin barrier.

Transdermal drug delivery has an advantage compared to systemic drug administration; this way, the drug is able to avoid the first-pass metabolism in the liver and the effect of an acidic environment in the stomach [24]. This “administration route” may also have special importance in those cases when the drug candidate shows instability in the plasma or the target tissue is the skin itself. To the best of our knowledge, skin penetration of α-aminophosphonates has not been investigated yet in diffusion chambers, neither ex vivo nor in vitro [25,26,27]. There are only data available from in silico studies on the gastrointestinal tract, blood-brain barrier and skin models [18,28].

The aim of this study was to uncover whether the topical semisolid formulation of three preselected α-aminophosphonates (representative molecules from a recently synthesized group, structures **1a**, **1b** and **1c**), are (1.) able to cross the artificial cellulose-acetate membrane (release), and (2.) able to penetrate through the ex vivo skin barrier in Franz diffusion cells or a skin-on-a-chip microfluidic system (absorption). These data may contribute to the early mapping of absorption and distribution profiles and medicinal chemistry development of potential lead compounds in this class of organic P-containing molecules.

## 2. Materials and Methods

### 2.1. Chemical Synthesis of α-Aminophosphonates

A mixture of 1.7 mmol (0.18 mL) benzylamine, 1.7 mmol substituted benzaldehyde (benzaldehyde: 0.18 mL; 4 methylbenzaldehyde: 0.20 mL; 4-chlorobenzaldehyde: 0.24 mL) and 1.7 mmol (0.22 mL) diethyl phosphite was irradiated in a sealed tube in a CEM Microwave reactor equipped with a pressure controller at the temperatures and for the times shown in Table 1. The volatile components were removed under reduced pressure. The residue so obtained was purified by flash column chromatography using silica gel and 3% MeOH in CH_2_Cl_2_ as the eluent to afford α-aminophosphonates **1a**–**c** as oils in purities of ≥98%. For identification data, see Table 2.

### 2.2. Drug Formulations, Particle Size Distribution and Rheology

α-Aminophosphonates were dispersed in the following hydrophilic cream base (O/W emulsion ointments) [32,33]: 46 m/m% Petrolatum ointment base, 10 m/m% Propylene glycol (Hungaropharma Zrt., Budapest, Hungary), 4 m/m% Paraffin oil (Hungaropharma Zrt., Budapest, Hungary), 38 m/m% Purified water. The components of the Petrolatum ointment base were 26 m/m% Vaselinum album (Hungaropharma Zrt., Budapest, Hungary), 12 m/m% Cetostearyl alcohol (Molar Chemicals Kft, Halásztelek, Hungary), 10 m/m% Propylene glycol, 8 m/m% Paraffin oil, 4 m/m% Polyoxyethylene sorbitan monostearate (Poly-sorbate 60) (Hungaropharma Zrt. Budapest, Hungary), and 40 m/m% Purified water (Table 3). The concentration of propylene glycol was set based on the recommendations on topical dosage forms [34].

The particle size distribution was measured with a Malvern Mastersizer2000 (Malvern Instruments Ltd., Malvern, UK) laser diffraction device. In total, 1 g of sample was dispersed in 10 mL of water, and after 1 min of Vortex shaking, 1 mL was taken and added to the sample dispenser unit containing 100 mL of purified water. The dispenser unit was applied at 1500 RPM.

The multi-channel detector system measured the scattering of red laser light with a wavelength of −632.8 nm, and the evaluation software provided the results after three parallel exposures according to the Fraunhofer method. The average curves were calculated from three parallel measurements.

Microscopic images were taken on Keyence VHX 970 digital microscope (VH-Z20R lens, magnification: 200×) (Mechelen, Belgium) to observe the structure of the semisolid formulations.

Rheological measurements were performed by Kinexus Pro Rheometer (Malvern Instruments Ltd., UK), registering the data with rSpace for Kinexus Pro 1.3 software. Semisolid samples were characterized using a cone and plate geometry where the gap for sample placement was 0.15 mm. Rotational measurements of formulations were determined at 25 °C controlled with an accuracy of ±0.1 °C by Peltier system of the instrument. In all measurements, a cylindrical cover made of stainless steel was placed over the samples in order to create a closed, saturated volume around the sample and prevent evaporation of the sample.

### 2.3. Membranes and Skins

For the in vitro studies, cellulose acetate filter with pore size of 0.45 μm (Sartorius AG, Göttingen, Germany) was cut to size (35 mm and 18 mm diameter discs for Franz diffusion cell and skin-on-a-chip microfluidic system, respectively) and soaked in isopropyl myristate (Molar Chemicals Kft., Halásztelek, Hungary) for 30 min before the experiment. The excess of isopropyl myristate was removed, and the impregnated cellulose acetate membrane was placed into the Franz diffusion cell or microfluidic diffusion chamber.

In the ex vivo experiments, the abdominal skin of male Wistar rats (ToxiCoop, Budapest, Hungary), with 520–609 g bodyweight, were used. The animals had free access to food and water before the study. The experiments were performed in compliance with the guidelines of the Association for Assessment and Accreditation of Laboratory Animal Care International and were in accordance with the spirit of the license issued by the Directorate for the Safety of the Food Chain and Animal Health, Budapest, and Pest Agricultural Administrative Authority, Hungary (PE/EA/4122-7/2016). The rats were anaesthetized intraperitoneally with chloral hydrate (400 mg/kg). The abdominal surface was shaved with an electric shaver and epilated with an epilator cream (X-epil, Aveola Kft., Budapest, Hungary). The skin was washed, wiped dry and mechanically sensitized 10 times by tape stripping with leucoplast (BSN Medical GmbH, Hamburg, Germany) to remove the upper layers of dead keratinocytes. This technique enables reaching detectable concentrations in the dermis and hypodermis. The double-layered skin (pinched together by hand) thickness of the epilated tape-stripped skin was measured with an engineer’s micrometer (Asimeto, Budapest, Hungary). The average double-layered skin thickness was 1.53 ± 0.03 mm. After the first cutting with blunt Metzen scissors, connecting tissue, fat and blood vessels were carefully removed. For the Franz diffusion cell and skin-on-a-chip studies, full-thickness skin incisions were made by punches (Stubai GmbH, Fulpmes, Austria) in diameter of 35 mm and 18 mm, respectively. The excised samples were wrapped in aluminium foil and stored in a deep freezer at −80 °C. On the day of the study, the skin preparations were taken out from the freezer for 30 min before the initiation of the experiment and thawed at room temperature.

### 2.4. Drug Delivery Study in Franz Diffusion Cells on Synthetic Membranes and Excised Skins

**Figure 1:**
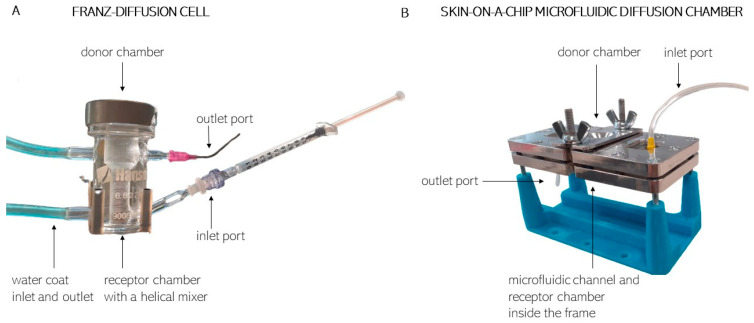
Techniques used in the penetration studies.

Hanson 6-Cell Manual Diffusion Test System-type vertical diffusion cells (Franz cell) were used (Hanson Research Corp., Chatsworth CA, USA; ABL&E-JASCO Hungary Kft., Budapest, Hungary, Figure 1A). Each unit was composed of a donor chamber, which was filled with 1000 µL of the aminophosphonate cream by a Microman E piston gel positive replacement pipette (Gilson, Middleton, WI, USA). The sample was layered in a uniform layer on the top of the membrane. The weight of the sample was measured with analytical accuracy. The receptor chamber contained peripheral perfusion fluid (PPF, consisting of 147 mM NaCl, 4 mM KCl and 2.3 mM CaCl_2_, all substances acquired from Sigma-Hungary Kft., Budapest, Hungary). A magnetic helical stirrer was placed in the acceptor chamber to enable the uniform distribution of the penetrated compound in PPF. The membrane or skin sample (with a diffusion surface of 1.767 cm^2^) was mounted between the two compartments.

In the case of each tested aminophosphonate, three units contained the artificial membrane, and three units had rat skin. The Franz cell units were tempered to 32 °C. During the experiment, 0.5 mL samples were taken every 30 min from the acceptor chamber, and the volume was replaced with PPF. The samples were collected for 5 h, placed immediately on dry ice and stored at −80 °C until bioanalysis.

### 2.5. Drug Delivery Study in Skin-on-Chip on Synthetic Membranes and Excised Skins

The skin-on-a-chip microfluidic diffusion chamber is a polydimethylsiloxane-based system in an aluminium frame (Figure 1B). Similar to the Franz diffusion cells, it contains two compartments, and the membrane or skin is placed between them. The major advantage of this technique is the reduction in needed volumes and pieces in the required components, membranes and skins, as discussed in detail in our previous papers [25,35]. The diffusion surface is 0.5 cm^2^, which separates the aminophosphonate cream-containing donor chamber and the PPF-filled receptor chamber. Contrary to the static Franz diffusion cells, the microfluidic diffusion chamber is a dynamic system, i.e., the PPF flow is continuous in the receptor compartment, ensured by a syringe pump (NE-1000, New Era, Farmingdale, NY, USA), which includes a 5 mL syringe filled with PPF, at a flow rate of 4 µL/min during the experiment. The PPF solution was running through the chip, filling the receptor chamber and the microfluidic channel and leaving the device at the outlet into collection vials. The samples were collected every 30 min for 5 h. The collected samples were placed on dry ice and stored at −80 °C until the bioanalysis. First, the experiments were performed at room temperature (23.5 °C) with temperature regulation by automatic air conditioning system in the lab in three parallels. The investigations were then repeated at 32 °C, putting the whole system into an incubator (Steriliser SN160, Memmert GmbH, Schwabach, Germany). As the volume capacity of incubator was low, only one syringe pump and skin-on-a-chip could be tested in the instrument at the same time.

### 2.6. Bioanalysis of α-Aminophosphonates

Mass spectrometric measurements were run on a Sciex 3200 QTrap mass spectrometer equipped with TurboV ion source operated in electrospray mode. A Perkin Elmer series 200 HPLC system was used for the separation.

A Perck PurospherStar 55 × 2 mm, 5 µm column was used in gradient elution mode. Water and acetonitrile, both containing 0.1% formic acid, were used as mobile phases. Flow rate was 500 µL/min. The gradient profile was A/B(time) 90/10(0.5)-3.5-5/95(1)-0.5-90/10(2.5). Injection volume was 5 µL. The mass spectrometer was operated in MRM mode. MRM transitions were **1a**: 334/196 (CE17), 334/91 (CE47); **1b**: 368/230 (CE19), 368/91 (CE45); and **1c**: 348/210 (CE19), 348/91 (CE51). The dwell time of each MRM transition was 100 msec. Source conditions were the following: curtain gas: 35 arbitrary unit (au); spray voltage: 5000 V; source temperature: 450 °C; and evaporating and drying gases were 40 and 40 au, respectively.

### 2.7. Statistical Analysis of the Data

The Franz diffusion cell data and the skin-on-a-chip data were averaged, and the means and standard error of the mean (SE) were calculated and presented. Area under the curve (AUC) values were calculated using Origin Pro software. For statistical comparison, 2-way ANOVA followed by Tukey’s post hoc test or Student’s t test were used. A *p* value of <0.05 was considered to indicate statistical significance.

## 3. Results and Discussion

**Scheme 2:**
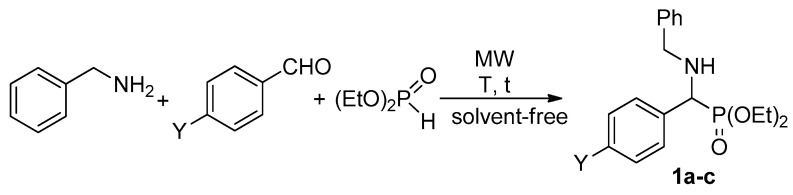
Synthesis of α-aminophosphonates by the Kabachnik-Fields reaction.

**Figure 2:**
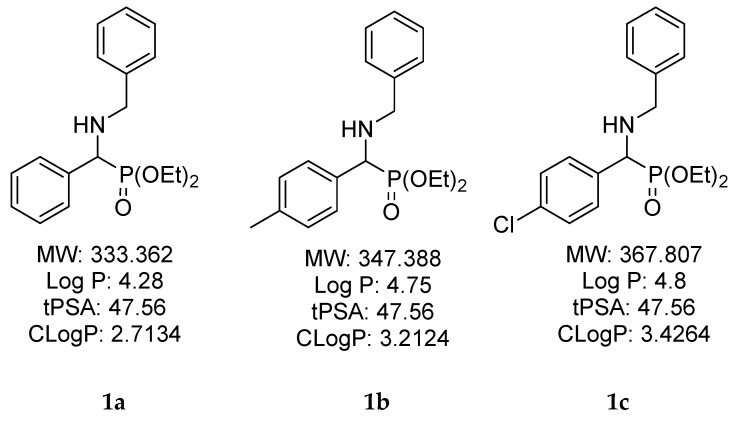
Chemical structure of the α-aminophosphonate derivatives applied in the experiments [30].

The bioactivity of aminophosphonates is the consequence of their structural similarity to amino acids. The compounds shown in Scheme 2 and Figure 2 were applied in the permeability tests. The tested compounds were prepared by reproductive synthesis based on our previous results [36,37,38]. The reactions of benzaldehyde derivatives, benzylamine and diethyl phosphite were carried out under microwave (MW) irradiation without the use of any solvent. α-Aminophosphonate **1a** obtained from benzaldehyde and diethyl phosphite at 100 °C after a 45 min irradiation was isolated in a yield of 85% (Table 1/Entry 1). Completion of the similar reaction of 4 methylbenzaldehyde required irradiation at 110 °C for 1.5 h to afford the corresponding product **1b** in 87% (Table 1/Entry 2). The analogous reaction of 4 chlorobenzaldehyde at 100 °C/40 min furnished aminophosphonate **1c** in a yield of 95% (Table 2/Entry 3). It is obvious that electron-donating substituents slow down the condensation. Table 2 summarizes the data required for the identification of α-aminophosphonates.

### 3.1. Droplet Size Distribution and Rheological Charactreization of the Creams

**Figure 3:**
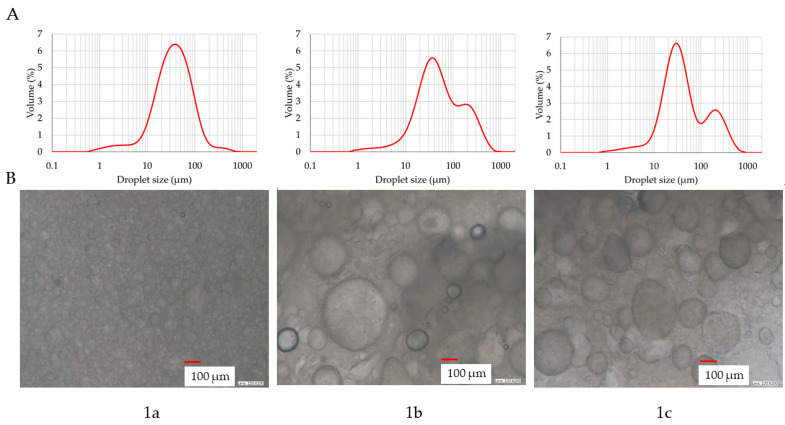
Particle size distribution in 2% cream formulation of **1a** left, **1b** middle, **1c** right, based on (**A**) laser diffraction method (Mastersizer 2000, Malvern, UK), (**B**) microscopic images (Keyence VHX 970 digital microscope, VH-Z20R lens, magnification: 200×, Mechelen, Belgium); red scale indicates 100 µm.

The particle size (droplet size) distribution in the 2% cream formulations of aminophosphonate **1a**, **1b** and **1c** compounds was determined (Figure 3). 1a molecule showed a homogenous distribution by size peaking at d = 33.156 µm with 7% of the volume. Molecule **1b** showed an inhomogeneous size composition presenting 2 peaks. One between d = 30–40 µm (about 6.3%) and a smaller one at d = 110 µm (about 3.2%). Compound **1c** showed also two peaks in particle size. One at d = 20–30 µm (7.3%) and the second one at d = 110 µm (~3.0%).

The microscopic examination of the cream formulations confirmed the particle size distribution showing homogenous droplet size distribution in sample **1a** and the presence of different sizes of particles in formulation **1b** and **1c** samples. The droplet size distribution was tested after 6 months, and no significant alteration was found in the characteristic d_0.1_, d_0.5_ and d_0.9_ values (SD < 0.05).

A possible explanation for the wider particle size distribution of molecules **1b** and **1c** can be the lower dispergation of the more lipophilic compounds (logP 4.8; 4.75). The excipient composition, sufficient to prepare a stable and homogenous O/W emulsion system from molecule **1a** (logP 4.28) with success, results in a less homogenous droplet size distribution in O/W emulsion semisolid preparation for molecules with a higher logP value.

**Figure 4:**
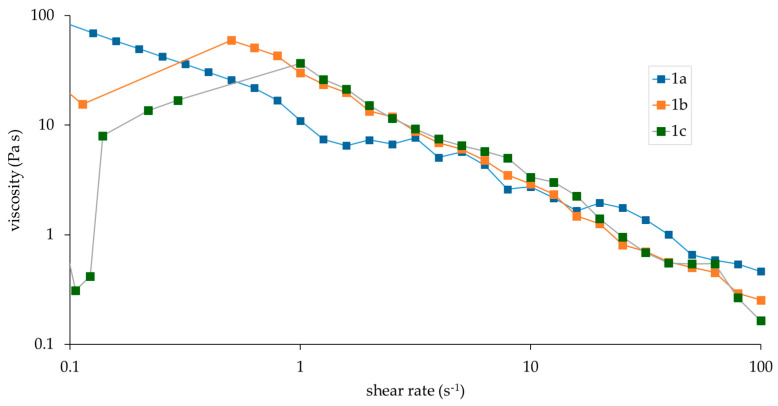
Viscosity curves of **1a**, **1b** and **1c** creams.

A rheological study was performed for the three creams at a 2% concentration (Figure 4). The viscosity curves show a proportional decrease in stress (e.g., increasing shear rate). The test results confirm the homogenous character of sample **1a** (molecule 1a). The viscosity changes at the lower shear rate are due to the relatively inhomogeneous structure of the formulations **1b** and **1c**. However, all the formulations showed a pseudoplastic (shear-thinning behaviour), where the viscosity values did not show much difference.

### 3.2. Drug Release and Penetration at 32 or 23.5 °C in Diffusion Cells

**Figure 5:**
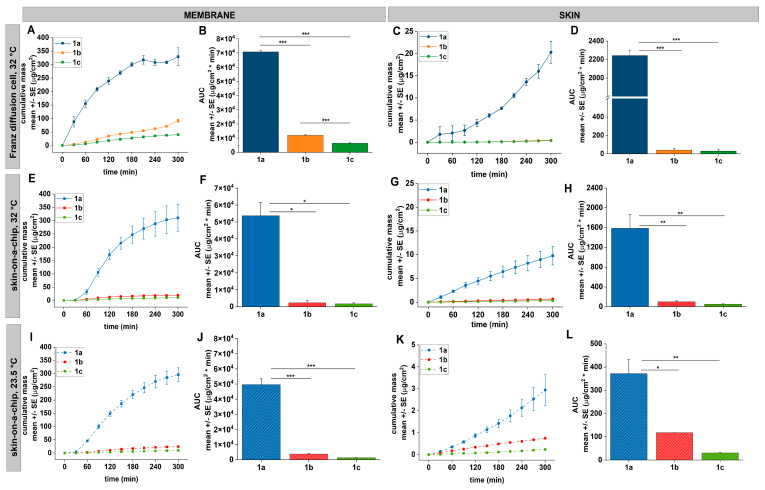
Release of three test compounds: **1a**, **1b**, **1c** from 2% cream formulation and penetration through the dermal barrier. Panels (**A**,**B**,**E**,**F**,**I**,**J**) show the cumulative mass-time curves and area under the curves (AUCs) of drug release from the semisolid topical formulation through cellulose acetate membrane studied in diffusion cells at 32 or 23.5 °C. Panels (**C**,**D**,**G**,**H**,**K**,**L**) show the cumulative mass-time curves and AUCs of drug penetration across the rat skin explants determined in diffusion cells at 32 or 23.5 °C, respectively. * *p* < 0.05, ** *p* < 0.01, *** *p* < 0.001, n = 3.

The three selected compounds were tested first in static Franz diffusion cells using artificial membranes (Figure 5A,B) or skin explants (Figure 5C,D). This method is to monitor the drug release from the formulation. The experiments were performed at 32 °C, which is similar to the physiological skin surface conditions. The diffusion of **1a** was the highest, and it showed rapid and saturating penetration kinetics in the membrane and an almost linear growing profile in rat skin. In the second part of the diffusion experiments, the three compounds were tested in physiologically more relevant conditions, in dynamic skin-on-a-chip diffusion cells (Figure 5E–L). In this device, a membrane or an excised skin was placed between the donor and acceptor chambers, and a continuous flow was generated below the membrane/skin by a syringe pump, mimicking the physiological microcirculation and shear stress in the tissue. The degree of the absorption of the molecules was lower than in Franz cells (in membranes: C_max_1a_ ~ 330 µg/cm^2^ in Franz cell vs. ~300 µg/cm^2^ in dynamic cell and in excised skins: C_max_1a_ ~ 21 µg/cm^2^ in Franz cell vs. ~9 µg/cm^2^ in dynamic cell), but the penetration order was in accordance with the static diffusion study showing the best permeability for **1a** compound. To study the effect of temperature on drug absorption, the dynamic diffusion 0065periments were repeated also at ambient temperature (23.5 °C) (Figure 5I–L). The diffusion profile in membranes was similar to the experiment at 32 °C, while on excised skins, the degree of skin penetration decreased largely in ambient temperature (C_max_1a_ ~ 9 μg/cm^2^ at 32 °C vs. ~2.8 μg/cm^2^ at ambient temperature). The higher temperature (32 °C) resulted in increased absorption in the ex vivo skins (Figure 5G,H vs. Figure 5K,L), as was expected, but no significant changes in the drug release through the artificial membrane (Figure 5E,F vs. Figure 5I,J). The difference in AUC values was statistically significant both in membranes and in skins between **1a** and the two other molecules.

Our previous study provided evidence that the permeability of excised abdominal rat skin is equivalent to that of the cellulose acetate membrane with a pore size of d = 0.45 µm [39]. In the current study, we used the same synthetic membranes. The much lower penetration of the compounds through the skin than through the membrane suggests that, besides the transappendageal absorption route, there are other, more important delivery routes in the skin (e.g., transcellular and paracellular) playing an important role in the molecular transport, which can be modulated at least partially by transporter proteins [40,41,42,43]. Further studies are needed to identify whether these APP molecules are substrates or inhibitors of efflux or uptake transporters in various skin cells (keratinocytes, fibroblasts, melanocytes and vascular cells).

### 3.3. Effect of Temperature on Drug Diffusion

**Figure 6:**
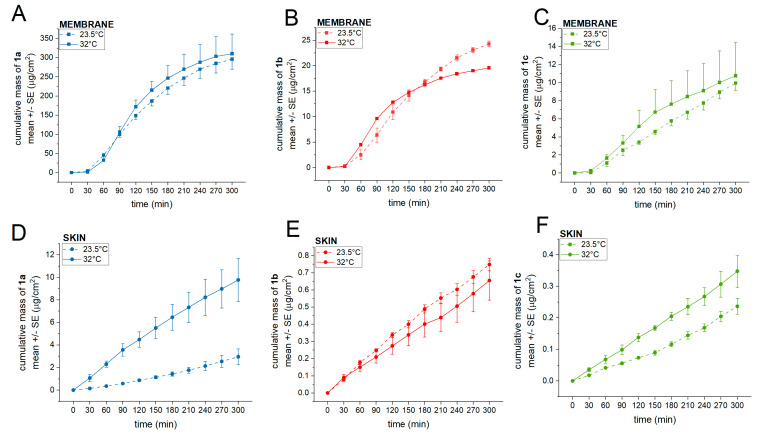
Cumulative mass-time profiles of three test compounds: **1a**, **1b**, **1c** released (**A**–**C**) or penetrated (**C**,**D**,**F**) from 2% cream formulation in vitro and ex vivo. (**A**,**D**) compound **1a**, (**B**,**E**) compound **1b**, (**C**,**F**) compound **1c** at skin surface temperature (32 °C) and at ambient temperature (23.5 °C) measured either in cellulose acetate membrane or ex vivo excised skins. Comparative study in dynamic diffusion cells.

The comparison of the drug diffusion at ambient temperature (23.5 °C) and at 32 °C in membranes (Figure 6A–C) and skins (Figure 6D–F) is shown in Figure 6. The higher temperature only moderately enhanced the penetration through the membranes (**1a**) but significantly improved the absorption of **1a** and **1c** across the excised skins (Figure 6D,F). Interestingly, **1b** diffusion was practically independent of the temperature (Figure 6E) at the studied temperatures.

## 4. Conclusions

In the current study, three previously synthesized α-aminophosphonates were tested for their transdermal penetration characteristics in traditional diffusion cells and in the skin-on-a-chip device in excised rat skin.

Excised human skin is utilized for in vitro permeation experiments to evaluate the safety and effect of topically-applied drugs by measuring their skin permeation and concentration. However, ethical considerations and limited availability are the major problems for using human skin to evaluate percutaneous absorption. Moreover, large variations have been found among human skin specimens as a result of differences in gender, age, race and anatomical donor site. Animal skins can be better standardized; these samples are frequently used to predict the in vivo human penetration/permeation of topically-applied chemicals. Although there are differences in the structure and thickness of rodent and human skins [44], a fairly good relationship was observed between the permeability coefficient between human and rat skin or cultured human skin models when in vitro skin permeation experiments were conducted in appropriate conditions, such as without excessive skin hydration and without permeation enhancers [44]. In the current experiments, no penetration enhancers and excessive hydration were added to the APP formulations.

In our recent study, the three preselected molecules were investigated for antitumor properties in a multidrug-resistant (MDR) uterine sarcome cell line [30]. Regarding the degree of cytotoxicity, the most effective compounds were **1b** and **1c**, while the unsubstituted **1a** molecule was less cytotoxic. As compared to other cancer cell lines, the MDR cells were the most susceptible to the cytotoxicity of the α-aminophosphonates, indicating that no or little P-glycoprotein interaction is present between the cells and our molecules.

Based on the results of this study, both the release from the formulation and the penetration across the dermal barrier were the highest in the case of unsubstituted **1a** molecule, while 4-MePh and 4-ClPh species only poorly penetrated through the skin. The skin absorption was temperature-dependent, mainly in the case of species **1a**. Furthermore, the particle size and rheological studies revealed that the 2% cream comprising substrate **1a** was the most homogenous formulation. The differences among the APPs tested in transdermal delivery can be explained by the variation in their lipophilicity. The most hydrophilic compound was **1a** (logP = 4.28 and cLogP = 2.7), while the 4-MePh, **1b** (logP = 4.75 and clogP = 3.21) and 4-ClPh, **1c** (logP = 4.8 and clogP = 3.43) showed more lipophilic character. The main penetration routes of the **1a** compound may involve transappendageal and transcellular pathway elements. Based on our data, it can be supposed that in the transcellular route, an absorptive transport mechanism is involved in the penetration process. This should be clarified in a later study. Moreover, the smaller and more homogenous droplet size and distribution in the case of **1a** molecule (d = 33 μm) can be responsible for better passage through the dermal barrier.

These observations indicate that topical delivery of the selected three α-aminophosphonates is feasible. The unsubstituted compound showed rapid and strong transdermal absorption both in static and dynamic conditions. For optimization of the pharmacological and pharmacokinetic properties and to improve the topical delivery of the more effective analogues, further chemical/technological modifications and in vivo PK/PD studies are needed. The efflux and uptake transporter interactions of the lead molecules should also be tested in transporter-specific assays [40,41,42,43].

## Figures and Tables

**Table 1:** Identification data of the three α-aminophosphonates **1a**–**c** selected as the starting material.

Entry	n	Y	T (°C)	t (min)	Yield (%)	Product
1	1	H	100	45	85	**1a**
2	1	4-Me	110	90	87	**1b**
3	1	4-Cl	100	40	95	**1c**

T: temperature of MW irradiation, t: irradiation time.

**Table 2:** Identification of the aminophosphonates synthesized.

Compounds	^31^P (ppm)	^31^P_lit_ (ppm)	Reference	[M + H]^+^	[M + Na]^+^
**1a**	23.5	23.7	[29]	334	356.1389, requires 356.1392 C_18_H_24_NO_3_PNa
**1b**	23.7	23.6	[30]	348	370.1548, requires 370.1548 C_19_H_26_NO_3_PNa
**1c**	22.9	22.9	[31]	368	390.1001, requires 390.1002 C_18_H_23_NO_3_PClNa

**Table 3:** The final excipient composition of 2% α-aminophosphonate creams.

Excipient	Concentration (%)	Function	Supplier
Paraffin oil	11.4	lipophilic base	Hungaropharma Zrt. Budapest, Hungary
Polyoxyethylene sorbitan monostearate (Polysorbate 60)	1.8	hydrophilic emulsifying agent	Hungaropharma Zrt. Budapest, Hungary
White petrolatum	12.0	lipophilic base	Hungaropharma Zrt. Budapest, Hungary
Cetostearyl alcohol	5.5	lipophilic emulsifying agent	Molar Chemicals Kft, Halásztelek, Hungary
Propylene glycol	14.6	antimicrobial preservative, stabiliser	Hungaropharma Zrt. Budapest, Hungary
Purified water	52.7		

## Data Availability

Raw data are available from the authors by request.
